# Asynchronous e-learning with technology-enabled and enhanced training for continuing education of nurses: a scoping review

**DOI:** 10.1186/s12909-023-04477-w

**Published:** 2023-07-13

**Authors:** Rika Kimura, Mayumi Matsunaga, Edward Barroga, Naoko Hayashi

**Affiliations:** 1 Department of Adult Health Nursing, Graduate School of Nursing Science, St. Luke’s International University, 10-1 Akashi-Cho, Chuo-Ku, Tokyo, 104-0044 Japan; 2 Department of Nursing, Faculty of Human Sciences, Sophia University, Tokyo, Japan; 3 Department of Medical Education, Showa University School of Medicine, Tokyo, Japan; 4 Graduate School of Nursing Science, St. Luke’s International University, Tokyo, Japan

**Keywords:** Asynchronous e-learning, Technology-enabled and enhanced training, Continuing education, Clinical nurses

## Abstract

**Background:**

Asynchronous e-learning has become the mainstream choice since the transformation of learning formats by the coronavirus disease-19 pandemic. This scoping review aimed to examine the technologies used in asynchronous e-learning for the continuing education of clinical nurses and their modes of delivery and effectiveness.

**Methods:**

This scoping review covered the period between 2011 and 2023. Six databases were searched for relevant studies following the Preferred Reporting Items for Systematic Reviews and Meta-Analysis extension for Scoping Reviews (PRISMA-ScR) protocol.

**Results:**

Sixty articles met the inclusion criteria. There was a noticeable trend toward using diverse technology-enabled and enhanced training (TEET) options after 2017. The enabling technological approaches, such as interactive online modules (25 articles) and video modules (25 articles), are described in the articles. The most commonly used enhancing technologies were scenario-based learning (nine articles), resource access (eight articles), computer simulation or virtual reality (three articles), and gamification (three articles). Among the outcomes, knowledge acquisition was the most commonly examined outcome (41 articles).

**Conclusions:**

Notably, many interactive TEET modules were used in asynchronous e-learning. There were few studies on gamification, computer simulation or virtual reality, and scenario-based learning (techniques to enhance intrinsic motivation further). However, the adoption of asynchronous e-learning with advanced TEET options is anticipated to increase in the future. Therefore, objective outcome measures are required to determine the effects of such learning methods on knowledge acquisition and behavioral changes.

## Background

E-learning enables learning regardless of the learner’s geographic location and time [1, 2]. In recent years, it has become the mainstream choice for continuing education for nurses, and changes in learning formats prompted by the coronavirus disease 2019 (COVID-19) pandemic have further increased the uptake of e-learning [3–5]. E-learning can be synchronous, connecting learners with instructors and other students in real-time, or asynchronous, allowing learners to study at a time and place of their choice [6]. The asynchronous type is a learner-directed method suitable for adult learning which enables learners to balance professional development with personal and professional obligations, particularly for nurses with irregular work schedules [7, 8]. However, because of the high level of independence among learners using e-learning, the lack of motivation for learning is considered a serious issue, and various types of asynchronous e-learning models have been developed to overcome this challenge.

The asynchronous e-learning models and designs reported in the literature range from slide-based models to types that include interactive elements [9]. Delivery methods also vary, with the most common methods categorized as (a) *enhanced or supplemental*, serving as an aide to face-to-face classroom learning and providing students with relative independence; (b) *blended e-learning models*, integrating face-to-face classroom and online learning; and (c) *pure online or fully online models* that provide students with maximum independence with no classroom or traditional face-to-face learning [10]. In recent years, novel e-learning methods, such as gamification and augmented or virtual reality (AR or VR), have been developed using information communication technology (ICT) and other technologies [1, 11, 12].

The term technology-enabled and enhanced training (TEET) reflects the various contributions of technology to education [13]. TEET includes both technology-enabled and technology-enhanced training. Technology-enhanced training can improve the effectiveness of interactive learning using videos, graphics, images, or simulations [14]. TEET continues to evolve as it is closely aligned with technological advancements [14]. However, very few studies have examined the effectiveness of e-learning using innovative technology in continuing education for nurses [1].

Button et al. [15] conducted a literature review to identify the technologies used for e-learning and ICT in nursing education and the problems the learners and educators face. The results revealed issues related to learners’ e-learning, information technology use, educators’ pedagogy, workload, and staff development for e-learning and related technologies. However, this study was a 10-year-old literature review covering the period between 2001 and 2012, which differs from the current ICT situation, although the low level of computer literacy is still an issue. In addition, the e-learning format was simple, such as a PowerPoint slide format or simple video. Furthermore, because the target audience included clinical nurses and students, the characteristics of continuing education for health personnel, such as motivation to learn and outcome evaluation, were unclear.

Ngenzi et al. [14] conducted a scoping review to identify available and effective TEET options to provide continuing professional development to health care providers in Rwanda, a low-income country with a limited and widely distributed health workforce. Technologies were categorized into *modes of delivery *and* technological approaches*. The *technological approaches* are divided into two subcategories: *enabling technologies* and *enhancing technologies*. They found several valid TEET options for both pure e-learning and blended learning modes and internet-based technologies. In this previous review, all studies using technological approaches also measured changes in the health personnel’s knowledge, skills, and behaviors, leading to increased knowledge acquisition, skills and self-efficacy, and leadership skills [14]. However, this previous study concerns e-learning and ICT-based education. Therefore, with the current development of various novel asynchronous e-learning methods, we considered that by categorizing asynchronous e-learning in continuing education for clinical nurses within the framework of TEET options used by Ngenzi et al. (2021) [14], we could identify effective TEET options and their delivery methods and educational effectiveness. This scoping review is anticipated to provide suggestions regarding the features that can be included in asynchronous e-learning, which is expected to accelerate in the future, and is foreseen to clarify the effects of asynchronous e-learning and learner motivation, which is an issue in asynchronous e-learning.

### Purpose and research question

This study aimed to identify the features of asynchronous e-learning for the continuing education of clinical nurses. The following research questions were addressed:

1. What technologies are used (TEET options) and their delivery modes in asynchronous e-learning for clinical nursing education?
2. What are the effective outcomes and outcome measures adopted in clinical nursing education using asynchronous e-learning?
3. What are the benefits and issues related to asynchronous e-learning?

### Definition of terms

#### Asynchronous e-learning

A type of e-learning that does not have a human facilitator and allows self-directed learning at a time and place of the learner’s choice.

#### Synchronous e-learning

A type of e-learning wherein a human facilitator is present, and the learning takes place in real time at a fixed time. Learners are often able to interact with the instructor and other learners.

## Methods

A scoping review was selected as this study’s methodology. A scoping review provides an overview (mapping) of a broad body of literature, allowing for a comprehensive survey of current research and identification of areas where research has not yet been conducted (research gaps) [16, 17]. The scoping review methodology was conceptualized by Arksey and O'Malley [18] in 2005 and was subsequently developed by Levac et al. (2010) [19] and the Joanna Briggs Institute [20]. In 2018, Tricco et al. published guidelines for reporting scoping reviews as an extension of the PRISMA statement (i.e., Preferred Reporting Items for Systematic Reviews and Meta-Analysis) [17] (PRISMA-ScR: PRISMA extension for Scoping Reviews). The present review was conducted following the PRISMA-ScR protocol. The review framework consisted of 5 steps: (1) defining the research question; (2) identifying relevant studies; (3) selecting the studies; (4) charting the data; and (5) collecting, summarizing, and reporting the results.

### Identifying relevant studies

The lead author (RK) and our university librarian devised the search strategy. The period covered was between 2011 and 2021. The following indexing databases were searched: PubMed, CINAHL, Cochrane Library, ERIC, Embase, and Ichu-shi Web. The inclusion criteria were intervention studies and practice reports on asynchronous e-learning in clinical nursing education written in English or Japanese. Notably, the same educational system training program provides post-graduate education for nurses and midwives in Japan. Therefore, midwives were included in the target population. The exclusion criteria were as follows: studies with no description of whether the e-learning method was synchronous or asynchronous or of the TEET option used, conference proceedings, and studies that included nursing students. The search terms used are listed in Table 1. The target population was clinical nurses and midwives; however, we decided to include cases in which nurses were part of the health care team. Therefore, the search terms were not limited to nurses but also included health personnel. With the expert help of our librarian, we checked each database for the inclusion of nurses in the subterms of health care professionals. In addition, many studies did not specify whether the type of e-learning was synchronous or asynchronous; therefore, we searched extensively for terms relating to e-learning. The Patient, Concept, Context (hereafter, “PCC”) framework shown in Table 1 was used as follows; *Patient*: Health personnel, *Concept*: Asynchronous e-learning and TEET, and *Context*: Clinical setting. The search string was created by connecting search terms related to each PCC category with OR and combining them with AND. The search formula used in PubMed was as follows:

- ((“e-learning”[Title/Abstract] OR “electronic learning”[Title/Abstract] OR “web based learning”[Title/Abstract] OR “online-learning”[Title/Abstract] OR “ICT”[Title/Abstract]　OR “distance learn*”[Title/Abstract] OR “computer assisted instruction”[MeSH Terms] OR “internet based learning”[Title/Abstract] OR “technology enhanced learning"[Title/Abstract]) AND (“health personnel”[MeSH Terms] OR “health professional*”[Title/Abstract] OR “nurse”[Title] OR “nurses”[Title] OR “midwi*”[Title]) AND (“education, continuing”[MeSH Terms]).

**Table 1:** Search terms

Patient, Concept, Context (PCC)	Target	Search words
P: Patient	Health personnel	・ health personnel ・ health professional
(C) Concept	Asynchronous e-learning with technology-enabled and enhanced training	・ e-learning ・ electronic learning ・ web-based learning ・ online learning
(C) Context	Clinical setting	・ education ・ continuing education

We searched each database with the librarian, checking each subword so that the other databases would follow the same search formula.

The search was conducted on February 18, 2022, with a follow-up search on June 12, 2023, to add new literature. The Rayyan software was used to manage the search results.

### Selection of studies

**Fig. 1:**
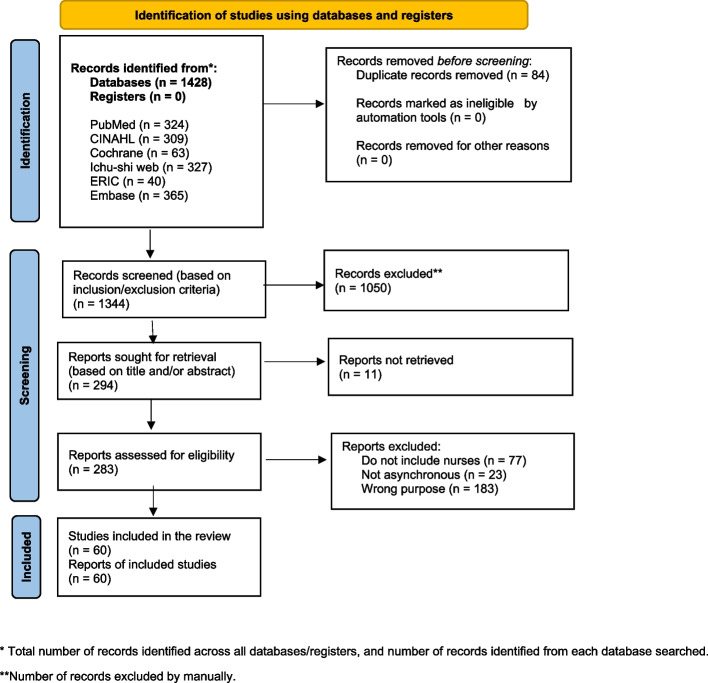
PRISMA-ScR flow diagram of the article selection process. * Total number of records identified across all databases/registers, and number of records identified from each database searched. **Number of records excluded by manually

An overview of the article selection process is presented in the PRISMA-ScR flow diagram (Fig. 1). In total, 1428 articles were selected, and after removing duplicate references using automation tools, 1344 articles were selected for screening. In the first stage, two researchers (RK and MM) independently and manually screened the titles, abstracts, and inclusion or exclusion criteria. The authors were blinded to each other’s judgment. They classified the studies using Rayyan as included, excluded, or undecided, and the authors discussed the articles classified as “conflicting” and “undecided.” The screening process yielded 294 studies, of which 283 were eligible for full-text review. After excluding 11 that were unavailable, a second screening was conducted similarly. Consequently, studies that did not include nurses in the target population (*n* = 77), did not focus on asynchronous e-learning (*n* = 23), or had different objectives (*n* = 123) were excluded, and 60 reports were finally selected (Table 2).

**Table 2:** Summary of the selected articles (Excel)

Author(s)	Year	Country	Participants (sample size)	Design	Objective(s)
Dennison [21]	2011	US	Nurses (*n* = 50)	Pre-post test design	To evaluate the computer-assisted learning module used in this study and its impact on its participants
Sherriff and Burston [22]	2012	Australia	Nurses (*n* = 107)	Quasi-experimental design	To evaluate the effect of an online medication calculation education and testing program
Spiva et al. [23]	2012	Canada	Nurses (*n* = 135)	Pre-post test design	To evaluate the effectiveness of nurses' ability to interpret basic electrocardiogram strips accurately using different learning modalities
Gordon et al. [24]	2013	US	Practicing pediatric respiratory therapists (RTs) (*n* = 40), registered nurses (RNs) (*n* = 163), and nurse practitioners (NPs) (*n* = 12)	Randomized controlled trial	To determine whether a brief educational video administered online improved nurse practitioners' knowledge, attitudes, and behaviors regarding skin cancer and skin cancer prevention counseling
Alipour et al. [25]	2014	Iran	Nurses ( *n* = 60)	Pre-post test design	To compare the effect of traditional face-to-face teaching with that of electronic learning via short message service (SMS) in improving the knowledge of nurses about breast cancer screening as part of the continuing education programs
Kim and Shin [26]	2014	Korea	Nurses (*n* = 32)	Pre-post test design	To test the effectiveness of an online problem-based learning (e-PBL) program that offers multimedia scenarios to develop sexual health care competencies
Liu and Chu [27]	2014	China	Nurses (*n* = 40)	Pre-post test design	To evaluate the nursing cace management e-learning program
McCrow et al. [28]	2014	Australia	Nurses (*n* = 147)	Cluster randomized controlled trial	To evaluate the impact of a delirium-specific educational website
Yoshikawa [29]	2014	Japan	Nurses (*n* = 32) (22 general nurses and 9 dysphagia nurses)	Prospective, descriptive study	To determine the effectiveness of interactive e-learning materials on the use of syringe pumps compared to traditional video materials
De Gagne et al. [30]	2015	Korea	Community health nurses (*n* = 25)	One-group, pretest–posttest design	To develop an online continuing education course on continence care for community health nurses and to examine its effectiveness
Delaney et al. [31]	2015	US	Critical care and emergency department nurses (*n* = 82)	Pre-post test design	To evaluate the impact or influence of a multimodal sepsis educational program for critical care and emergency department nurses on knowledge acquisition and self-assessed competence in the early recognition and treatment of patients with sepsis
Hsu et al. [32]	2015	China	Nurses (*n* = 104)	Pre-post test design	To evaluate the effect of an online caring curriculum in enhancing the nurses' caring behavior
Johnson et al. [33]	2015	Australia	Nurses (*n* = 71)	Pre-post test design	To investigate the impact of an e-learning education program for nurses on falls risk screening, falls prevention, and post-falls management
Kato et al. [34]	2015	Japan	Midwives (*n* = 48)	Pre-post test design	To evaluate an e-learning educational program for midwives to acquire knowledge about postpartum hemorrhage
Murphy et al. [35]	2015	UK	Healthcare professionals (*n* = 43)	Qualitative methodology (focus group interviews)	To develop and evaluate the efficacy of a freely available, internet-based learning resource for nurses and allied health professionals who provide nutrition, diet, and lifestyle advice for cancer survivors
Sarabia-Cobo et al. [36]	2015	Spain	Health professionals (*n* = 12,400)	Retrospective and observational study	To describe and profile the satisfaction and knowledge of students enrolled in two MOOCs on clinical safety, offered by the Department of Nursing at the University of Cantabria in the academic years 2012–2013 and 2013–2014
Berggren et al. [37]	2016	Sweden	District nurses/registered nurses and general practitioners/ physicians working with home care (*n* = 140)	Observational cohort study	To evaluate the effectiveness of a continuing educational intervention on primary health care professionals' familiarity with information important to nutritional care in a palliative phase, their collaboration with other caregivers, and their level of knowledge about important aspects of nutritional care
Okuroğlu and Alpar [38]	2016	Turkey	Healthcare professionals (*n* = 50) (44 nurses and 6 midwives)	Pre-post test design	To develop and evaluate a web-based type 2 diabetes training program (WB-DEP) for healthcare professionals
Perrego [39]	2016	US	Perioperative staff members (RNs, RN first assistants, surgical technologists) (*n* = 66)	Pre-post test design	To determine whether online education for perioperative staff on regulated waste disposal is effective in bringing about change in regulated waste management
Sarna et al. [40]	2016	China	Nurses (*n* = 1386)	Prospective single-group design	To evaluate a web-based educational smoking cessation program on changes in the frequency of hospital-based nurses’ self-reported interventions to help smokers quit using the 5 As (i.e. Ask, Advise, Assess, Assist, Arrange), to reduce exposure to second-hand smoke, and to change attitudes about nurses’ involvement in tobacco control
Sinclair et al. [41]	2016	Australia	Healthcare professionals (HCPs)	Systematic review	To identify, appraise, and synthesize the best available evidence on the effectiveness of e-learning programs for healthcare professionals' behaviour and patient outcomes
Bond et al. [42]	2017	Australia	Nurses (*n* = 236), pharmacists (*n* = 70), doctors (*n* = 271)	Pre-post test design	(1) To determine health professionals’ experience and knowledge of clinical use of vancomycin,an antibiotic used for treatment of serious infections caused by methicillin-resistant Staphylococcus aureus (MRSA) and (2)to describe the design and implementation of a Web-based e-learning tool created to improve knowledge in this area
Glover et al. [43]	2017	US	Critical care and emergency department nurses (*n* = 82)	Quasi-experimental design (a randomized crossover study)	To describe a collaboration between private industry and a hospital to modify, implement, and evaluate a simulation-based blended PIVC insertion continuing education program for staff nurses
Kong et al. [44]	2017	Korea	Nursing staff (nurses and geriatric care assistants) (*n* = 122)	Cluster-randomized controlled trial	To evaluate the effects of a multicomponent restraint reduction program (MRRP) for nursing staff in Korean nursing homes
Mannning et al. [45]	2017	UK	Nurses (*n* = 98)	Prospective, uncontrolled, intervention study/ mixed-methods, quasi-experimental design	(1) To determine the impact of a digital educational intervention on the knowledge, attitudes, confidence and behavioral intention of registered children's nurses working with children and young people (CYP) admitted with self-harm; (2) to explore the perceived impact, suitability, and usefulness of the intervention
Micheel et al. [46]	2017	US	Health care professionals (*n* = 751)	Pre-post test design	To assess learning styles of oncology healthcare professionals and to determine whether learning style-tailored educational materials lead to enhanced learning
Shin et al. [47]	2017	Korea	Nurses (*n* = 50)	Non-equivalent control group pretest–posttest design	To evaluate the effects of using e-learning on neurologic assessment knowledge, ability, and self-confidence among nurses
Smith et al. [48]	2017	US	Nurses (*n* = 34)	Pre-post test design	To evaluate the effect of a multimodal educational strategy on critical care nurses’ knowledge and confidence to assess and manage delirium using the CAM-ICU
Trudeau et al. [49]	2017	US	Primary care providers (*n* = 238)	Randomized controlled trial	To improve pain management practices, they developed an online interactive continuing education (CE) program for primary care providers (PCPs) and tested the efficacy of this program
Williams et al. [50]	2017	US	Nursing home staff (nurses and nursing assistants, speech/music/occupation therapist, ancillary staff) (*n* = 141)	Quantitative study / pre- post-and follow-up survey	To establish feasibility and determine the preliminary effects of the online program in preparation for a national pragmatic clinical trial
Ylönen et al. [51]	2017	Finland	Nurses ( *n* = 946)	Pre-post test design	To test the effectiveness of an Internet-based education programme about venous leg ulcer nursing care on perceived and theoretical knowledge levels and attitudes among nurses working in home health care
Goodman et al. [52]	2018	US	Nurse practitioners (*n* = 30)	Pre-post test design	To determine whether a brief educational video administered online improved nurse practitioners' knowledge, attitudes, and behaviors regarding skin cancer and skin cancer prevention counseling
Meredith et al. [53]	2018	Australia	Predominately nurses and occupational therapists (*n* = 121), including nurse (*n* = 38)	Multiple-method study employed a longitudinal survey design	To investigate the efficacy of this training when provided through a custom-designed e-learning package
Oneill et al. [11]	2018	US	Nurses (*n* = 37)	Pre-post test design	To increase nurses’ knowledge with respect to best practices for catheter associated urinary tract infections (CAUTI); to enhance frontline staff engagement in best practices using a technology-driven platform infused with game-based learning
Abel et al. [54]	2019	US	Nurses (*n* = 29)	Pre-post test design	To evaluate the effectiveness of an online learning, certificate programme for front-line nurse leaders' sense of empowerment
Gullatte et al. [55]	2019	US	Nurses and social workers (*n* = 20)	Pre-post test design	To measure the impact of TACE on improving the capability and comfort of caregivers with end-of-life communication
Kaneko et al. [56]	2019	Japan	Nurses (*n* = 26)	Non-randomized pre–post test design	To conduct an educational program based on the cognitive restructuring method in order to provide nurses with training in emotional coping, and to verify changes in emotional coping tendencies
Lineker et al. [57]	2019	Canada	Various professions in primary care, including family physicians, physiotherapists, occupational therapists and nurses (*n* = 89)	Pilot study	To evaluate whether the Arthritis Clinical Practice Guidelines GRIP online program improves primary care providers' recommendations and adherence to arthritis best practices and increases their confidence and satisfaction in their arthritis management capabilities
Rouleau et al. [9]	2019	Canada	Systematic review target of registered nurses (included study *n* = 22)	Systematic reviews of systematic qualitative, quantitative, mixed-studies reviews	To systematically summarize the qualitative and quantitative evidence regarding the effects of e-learning on nursing care among nurses in a continuing education context
Schilinski et al. [58]	2019	US	Nurses (*n* = 114)	One-group pretest–posttest, longitudinal study employing survey methodology	To determine if RNs retain and value education provided by an EDLM
Colaceci et al. [59]	2020	Italy	Healthcare professionals (*n* = 4582); most learners were nurses(*n* = 1820)	Pre–post test design	To evaluate the long-term effectiveness of an online national program on infant nutrition for HCPs
Harvey et al. [60]	2020	US	Health care professionals (most learners were nurses (75.19%) and a majority of learners worked in oncology (74.68%))	Pre–post test design	To evaluate the Cancer Survivorship E-Learning Series for Primary Care Providers developed to address the need for cancer survivorship training and education for healthcare providers focused on primary care
Horiguchi et al. [61]	2020	Japan	Nurses (*n* = 164)	Intervention study	To investigate the educational methods using E-learning teaching materials that were utilized to improve nurses’ skills in promoting team-based diabetes medical care
Howard and Embree [62]	2020	US	Nurses (*n* = 49)	Quasi-experimental mixed-methods design	To examine whether an educational intervention can increase awareness and knowledge of incivility and bullying and enhance communication skills
Isoyama [63]	2020	Japan	Midwives (*n* = 25)	Quasi-experimental study/before-and-after evaluation study	To develop and evaluate training programs to improve understanding and awareness of perinatal family role acquisition among midwives
Kurotaki [64]	2020	Japan	Nurse manager (*n* = 72)	Randomized controlled design	To verify the effectiveness of an educational program developed to improve hospital nursing administrators’ ability to accept supporting nurses
Martinengo et al. [12]	2020	Singapore	RCTs for healthcare professionals in chronic wound management	Systematic review	To assess the effectiveness of digital education in improving healthcare professionals' knowledge, attitudes, practical skills and behaviour change on chronic wound management, and their satisfaction with the intervention
Mun and Hwang [65]	2020	Korea	Nurses (*n* = 56)	Randomized controlled design	To develop a web-based anticancer chemotherapy nursing course for clinical nurses and to examine its effectiveness in terms of job knowledge, self-efficacy, and nursing performance
Shchory et al. [66]	2020	Israel	Nurses and physicians (*n* = 433: 73% nurses, 27% physicians)	Pre–post design	To explore the effect of an intervention program on the knowledge and attitudes among physicians and nurses regarding ADRs reporting
Yeo et al. [67]	2020	Singapore	Healthcare professionals who attended the Neonatal Resuscitation Course (mainly physicians and nurses) (*n* = 162)	Quantitative study / post-and follow- up survey	To report findings of a study on the effectiveness of a web-based game as an aid for retention of knowledge and technical skills in neonatal resuscitation subsequent to "standard" simulation-based training in neonatal resuscitation
Yoshida et al. [68]	2020	Japan	Nurse (*n* = 32) (22 general nurses and 9 dysphagia nurses)	Prospective, descriptive study	To clarify the effectiveness of an education program concerning the use of ultrasonography to assess swallowing function (the "Swallowing Point-of-Care Ultrasound Education Program")
Lim and Yi [69]	2021	Korea	Nurses (*n* = 118)	Randomized controlled trial	To develop a web-based education program using medical malpractice cases and to evaluate the effectiveness with regard to legal obligations and patient safety competency of nurses
Matsumoto et al. [70]	2021	Japan	Nurse educators (*n* = 38)	Pre–post test design	To report on a POCUS train-the-trainer program for nurse educators that targets lower urinary track dysfunction
Ota [71]	2021	Japan	Nurses (*n* = 16)	Intervention study	To develop an e-learning program on "support mother-infant attachment formation in the early postpartum period" for nurses engaged in early postpartum mother-infant care and to evaluate its usability (hereinafter referred to as UI)
Williams et al. [72]	2021	US	Nursing home staff (nurses and nursing assistants, speech/music/occupation therapist, ancillary staff) (*n* = 141)	Quantitative study/pre–post and follow-up survey	To establish feasibility and determine the preliminary effects of the online program in preparation for a national pragmatic clinical trial
Evelyn et al. [73]	2022	US	Nurses (*n* = 34)	Pre–post test design	To assess the overall nursing knowledge of the pain mechanisms of Sickle Cell Disease and self-reporting of pain and to measure the change in nursingknowledge and simulated practice behavior after completion of education focused on practice change
Fang et al. [74]	2022	Switzerland	Healthcare professionals caring for old patients and family members(*n* = 38)	Pre–post test design	To test the efectiveness of an intervention (the online communication skills training on trasitional care [OTCCST] and traditional care)
Nakamura et al. [75]	2022	Japan	Nurses ( *n* = 130)	Randomized controlled design	To evaluate a hiesho intervention program for nurses called "Preventing Hiesho: An Intervention Program for Education Nurses" in terms of its effectiveness in improving the knowledge and perception of nurses about the importance of hiesho care
Suzuki et al. [76]	2022	Japan	Nurses ( *n* = 71)	Pre–post test design	To evaluate the effectiveness of a "Dementia nursing practice competency development program" for nurses in acute care hospitals
Bos et al. [77]	2023	Netherlands	General practitioners (*n* = 17) and nurses(*n* = 16)	Pre–post test design	To evaluate a newly developed blended learning programme for general practitioners (GPs) and nurses in supporting shared decision making (SDM) about palliative cancer treatment in a simulated setting

### Charting the data

Data were extracted from the 60 articles, and data were charted for the following items: author, publication year, country, target population, study design aims, type of e-learning, outcomes, measurement tool of outcomes, benefits or effects related to e-learning technology, and issues or needs related to e-learning technology. The e-learning modes of delivery and technologies used were classified into modes of delivery and technological approaches (i.e., TEET) using the classification of Ngenzi et al. (2021) [14]. The quality of the articles was not examined as this step was not part of the study’s objective.

### Collating, summarizing, and analyzing data

A table summarizing the articles’ characteristics and findings was prepared, and a list of articles was compiled. An overview of the studies was conducted by systematically counting the geographic distribution of the articles, year of publication, TEET options, outcomes, and content analysis of the studies to identify the benefits, effects, and challenges related to e-learning technologies. The results were shared among the researchers, and the classification and results of content analysis were discussed to ensure consensus on the perceptions. Content analysis was conducted using the conventional content analysis method by Hsieh and Shannon (2005) [78]. The article’s content was read and summarized in the first stage according to semantic units (primary codes). In the second stage, primary codes were grouped and converted into secondary codes in light of the research objectives and analysis categories. In the third step, the codes were grouped into subcategories by comparing them from the viewpoint of similarity and difference. As for the fourth step, the codes were categorized based on the relationships among the subcategories.

## Results

### Characteristics of selected studies

**Fig. 2:**
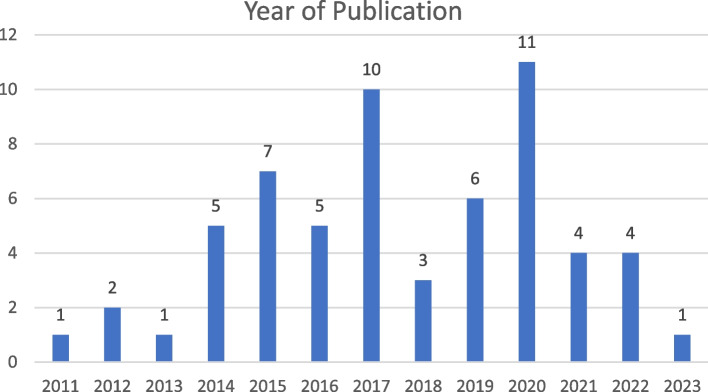
Year of publication

**Fig. 3:**
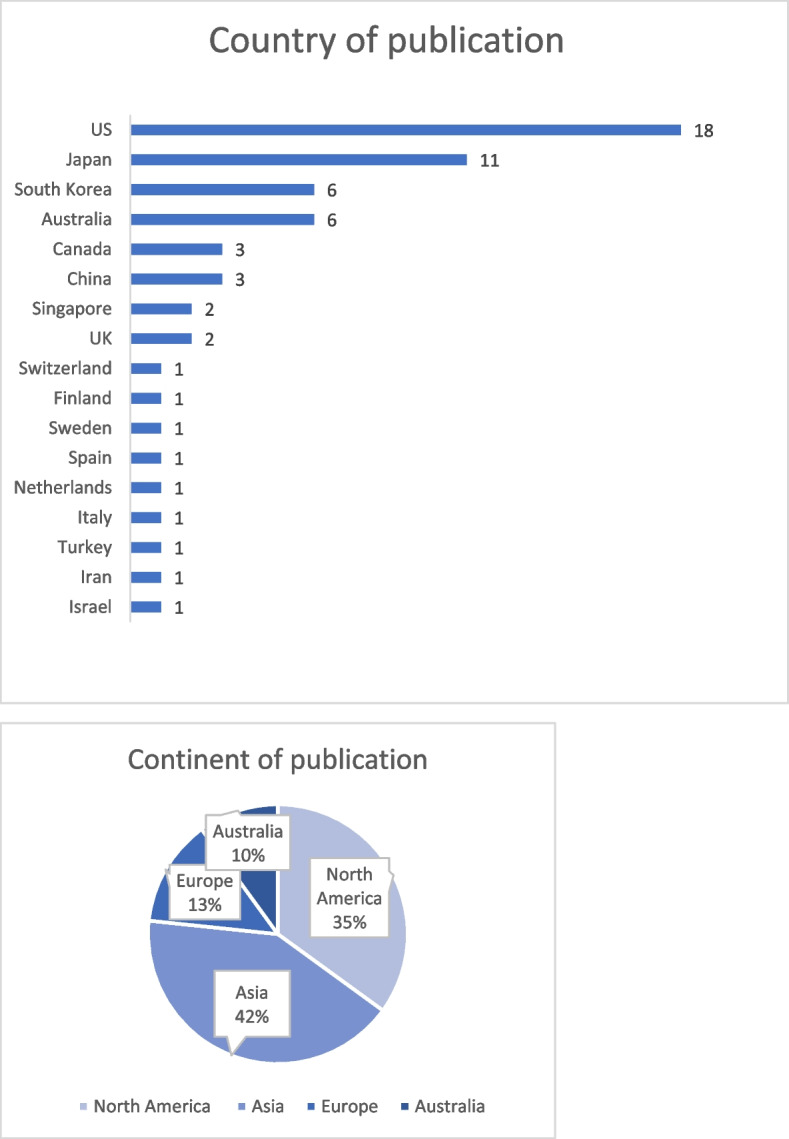
Country of publication

Between 2011 and 2023, 11 studies were published in 2020, 10 in 2017, and seven in 2015; 39 of the 60 studies were published in 2017 or later (Fig. 2). Data on country of publication are shown in Fig. 3. The most common continents of publication were Asia (25 articles: China, three [27, 32, 40]; Japan, 11 [29, 34, 56, 61, 63, 64, 68, 70, 71, 75, 76]; South Korea, six [26, 30, 44, 47, 65, 69]; Singapore, two [12, 67]; Turkey, one [38]; Israel, one [66]]; Iran, one [25]), followed by North America (21 articles: US, 18 [11, 21, 24, 31, 39, 43, 46, 48–50, 52, 54, 55, 58, 60, 62, 72, 73]; Canada, three [9, 23, 57]), Europe (eight articles: UK, two [35, 45]; Spain, one [36]; Italy, one [59]; Switzerland, one [74]; Netherlands, one [77]; Sweden, one [37]; Finland, one [51]) and Australia (six articles [22, 28, 33, 41, 42, 53]). Of the 60 studies, three were systematic review articles (Table 3) [9, 12, 41], while the remaining 57 were intervention studies. The study population in the 37 articles included only nurses, two included only midwives [34, 63], and 21 included health personnel, such as physicians and therapists. The total number of healthcare professionals included in the intervention studies examined in this study was 26,273. Of the study designs, the most common was pretest–posttest (*n* = 34), followed by quasi-experimental design (*n* = 8) and randomized controlled trial (*n* = 8), and systematic review (*n* = 3).

**Table 3:** Description of the reviews (Excel)

**Author**	**Technological Approach**													
	**Enabling Technology**								**Enhancing Technology**					
	DVD (Not Internet mediated)	Slides/ PowerPoint	Video	Online module /Interactive modules	Weblinks	MOOCs/ Webinar	Online discussion forum	Mobile device (Smart phone, digital tablet)	LMS (Learning Management System)	Narrarted PowerPoint	Computer simulation /virtual reality	Resource access (e.g. database, weblinks)	Scenario-based learning	Gamification
Sinclair et al. (2016) [41]		**レ**	**レ**							**レ**				
Rouleau et al. (2019) [9]				**レ**							**レ**			
Martinengo et al. (2020) [12]				**レ**		**レ**		**レ**	**レ**		**レ**			**レ**

**Author**	**Outcome that was effective**										
	Knowledge	Skills	Competency	Behavior/ performance	Attitude	Satisfaction	Confidence	Perceptions	Others	**Major findings**	**Issues and needs related to e-learning technology**
Sinclair et al. (2016) [41]				**レ**					**レ**(patient outcomes)	The results suggest that e-learning was at least as effective as traditional learning approaches, and superior to no instruction at all in improving health care professional behavior	Further research is needed to determine the effectiveness of asynchronous e-learning for behavior change using objective measurement scales; another objective scale based on sound theoretical constructs needs to be developed and validated to assess the behavioral outcomes of e-learning
Rouleau et al. (2019) [9]	**レ**			**レ**		**レ**				Most results were reported positively. For example, nurses were satisfied with the use of e-learning and improved knowledge	Nurse dissatisfaction with e-learning interventions was explained by the following reasons: technical difficulties, lack of computer experience and internet literacy, slow information exchange, and a preference for face-to-face format. In one SR, nurses identified access, navigation, and time as challenges
Martinengo et al. (2020) [12]	**レ**	**レ**		**レ**	**レ**	**レ**			**レ**(patient outcomes/cost)	Digital education on chronic wound management appears to be more effective than no intervention in terms of knowledge. Blended education on chronic wound management appears to be more effective than digital education in terms of knowledge and at least as effective in terms of skills	A high-quality research design that compares digital education to traditional face-to-face learning and other modalities is essential, for example, virtual simulation, virtual reality, and practical wound management skills training using augmented reality could be incorporated

### Technologies used (TEET options)

Table 4 shows the classifications of technological approaches based on the two categories mentioned by Ngenzi et al. (2021) [14]. The most commonly used technologies among enabling technologies were interactive online modules (25 articles) [11, 21–24, 28, 29, 31, 33, 35, 37, 42, 43, 45, 48–50, 53, 54, 56, 57, 62, 65, 66, 72] and videos (25 articles) [11, 24, 26, 30–32, 34, 35, 38, 42, 44, 50, 52, 57, 61, 65, 68, 70–77]. The next most commonly used technologies were slides or PowerPoint (11 articles) [11, 34, 39, 46, 47, 58, 59, 63, 69, 73, 77] and online discussions (seven articles) [11, 26, 28, 31, 54, 57, 72]. The most commonly used enhancing technology category was scenario-based learning (nine articles) [31, 32, 42, 51, 53, 54, 59, 62, 76], followed by resource access (eight articles) [24, 26, 28, 33, 35, 53, 59, 60] and narrated PowerPoint (eight articles) [23, 27, 46, 57, 60, 64, 71, 75], computer simulation or virtual reality (three articles) [31, 49, 62], and gamification (three articles) [11, 50, 67]. Five studies used multiple enhancing technology options [31, 53, 59, 60, 62]. Overall, there was a marked trend toward using various TEET options after 2017.

**Table 4:** Descriptions of the study mode of delivery, technological approach, and outcomes (Excel)

Author	Mode of Delivery		Technological Approach										
			**Enabling Technology**									**Enhancing Technology**	
	Blended learning	Pure online delivery	DVD (Not Internet mediated)	Slides/ PowerPoint	Video	Online module /Interactive modules	Weblinks	MOOCs/ Webinar	Online discussion forum	Mobile device (Smart phone, digital tablet)	LMS (Learning Management System)	Narrarted PowerPoint	Computer simulation /virtual reality
Dennison (2011) [21]		**レ**				**レ**							
Sherriff et al. (2012) [22]		**レ**				**レ**							
Spiva (2012) [23]		**レ**				**レ**						**レ**	
Gordon et al. (2013) [24]		**レ**			**レ**	**レ**							
Alipour et al. (2014) [25]		**レ**								**レ**			
Kim and Shin (2014) [26]		**レ**			**レ**				**レ**				
Liu and Chu (2014) [27]		**レ**	**レ**				**レ**					**レ**	
McCrow et al. (2014) [28]		**レ**				**レ**			**レ**				
Yoshikawa (2014) [29]		**レ**				**レ**							
De Gagne et al. (2015) [30]		**レ**			**レ**		**レ**						
Delaney et al. (2015) [31]		**レ**			**レ**	**レ**			**レ**				**レ**
Johnson et al. (2015) [33]		**レ**				**レ**							
Kato et al. (2015) [34]		**レ**		**レ**	**レ**								
Murphy et al. (2015) [35]		**レ**			**レ**	**レ**							
Sarabia-Cobo et al. (2015) [36]		**レ**						**レ**					
Berggren et al. (2016) [37]	**レ**					**レ**	**レ**						
Okuroğlu and Alpar (2016) [38]		**レ**			**レ**						**レ**		
Perrego (2016) [39]		**レ**		**レ**									
Sarna et al. (2016) [40]		**レ**					**レ**						
Bond et al. (2017) [42]		**レ**			**レ**	**レ**							
Glover et al. (2017) [43]	**レ**					**レ**							
Kong et al. (2017) [44]	**レ**				**レ**								
Hsu et al. (2015) [32]		**レ**			**レ**								
Mannning et al. (2017) [45]		**レ**				**レ**							
Micheel et al. (2017) [46]		**レ**		**レ**								**レ**	
Shin et al. (2017) [47]		**レ**		**レ**									
Smith et al. (2017) [48]	**レ**					**レ**							
Trudeau et al. (2017) [49]		**レ**				**レ**							**レ**
Williams et al. (2017) [50]		**レ**			**レ**	**レ**							
Ylönen et al. (2017) [51]	**レ**										**レ**		
Goodman et al. (2018) [52]		**レ**			**レ**								
Meredith et al. (2018) [53]		**レ**				**レ**							
Oneill et al. (2018) [11]		**レ**		**レ**	**レ**	**レ**			**レ**				
Abel et al.(2019) [54]	**レ**					**レ**			**レ**				
Gullatte et al. (2019) [55]		**レ**						**レ**					
Kaneko et al. (2019) [56]		**レ**				**レ**				**レ**			
Lineker et al. (2019) [57]		**レ**			**レ**	**レ**			**レ**			**レ**	
Schilinski et al. (2019) [58]		**レ**		**レ**									
Colaceci et al. (2020) [59]		**レ**		**レ**							**レ**		
Harvey et al. (2020) [60]		**レ**										**レ**	
Horiguchi et al. (2020) [61]		**レ**			**レ**								
Howard and Embree (2020) [62]		**レ**				**レ**							**レ**
Isoyama (2020) [63]	**レ**			**レ**									
Kurotaki (2020) [64]		**レ**								**レ**	**レ**	**レ**	
Mun and Hwang (2020) [65]		**レ**			**レ**	**レ**							
Shchory et al. (2020) [66]	**レ**					**レ**							
Yeo et al. (2020) [67]		**レ**											
Yoshida et al. (2020) [68]	**レ**				**レ**								
Lim and Yi (2021) [69]		**レ**		**レ**									
Matsumoto et al. (2021) [70]	**レ**				**レ**								
Ota (2021) [71]		**レ**			**レ**					**レ**	**レ**	**レ**	
Williams et al. (2021) [72]		**レ**			**レ**	**レ**			**レ**				
Evelyn et al. (2022) [73]		**レ**		**レ**	**レ**								
Fang et al. (2022) [74]		**レ**			**レ**								
Nakamura et al. (2022) [75]		**レ**			**レ**							**レ**	
Suzuki et al. (2022) [76]		**レ**			**レ**								
Bos et al. (2023) [77]	**レ**			**レ**	**レ**		**レ**						

Author				Outcome that was effective								
	**Enhancing Technology**											
	Resource access (e.g., database, weblinks)	Scenario-based learning	Gamification	Knowledge	Skills	Competency	Behavior/ performance	Attitude	Satisfaction	Confidence	Perceptions	Others
Dennison (2011) [21]				**レ**								
Sherriff et al. (2012) [22]				**レ**					**レ**			**レ**(self efficacy)
Spiva (2012) [23]				**レ**						**レ**		
Gordon et al. (2013) [24]	**レ**			**レ**			**レ**		**レ**			
Alipour et al. (2014) [25]				**レ**								
Kim and Shin (2014) [26]	**レ**			**レ**								
Liu and Chu (2014) [27]				**レ**						**レ**		
McCrow et al. (2014) [28]	**レ**			**レ**							**レ**	
Yoshikawa (2014) [29]				**レ**								
De Gagne et al. (2015) [30]				**レ**				**レ**	**レ**			
Delaney et al. (2015) [31]		**レ**		**レ**		**△**						
Johnson et al. (2015) [33]	**レ**						**レ**					
Kato et al. (2015) [34]				**レ**					**レ**			
Murphy et al. (2015) [35]	**レ**			**レ**			**レ**	**レ**				
Sarabia-Cobo et al. (2015) [36]									**レ**			**レ**(completion)
Berggren et al. (2016) [37]				**レ**								**レ**(collaboration with other caregivers)
Okuroğlu and Alpar (2016) [38]				**レ**					**レ**			
Perrego (2016) [39]				**レ**			**レ**					**レ**(cost)
Sarna et al. (2016) [40]								**レ**				
Bond et al. (2017) [42]		**レ**		**レ**			**レ**			**レ**		
Glover et al. (2017) [43]				**レ**	**レ**					**レ**		
Kong et al. (2017) [44]				**レ**				**レ**			**レ**	
Hsu et al. (2015) [32]		**レ**					**レ**	**レ**				
Mannning et al. (2017) [45]				**レ**			**レ**	**レ**		**レ**		
Micheel et al. (2017) [46]				**レ**								
Shin et al. (2017) [47]							**レ**					
Smith et al. (2017) [48]										**レ**		
Trudeau et al. (2017) [49]				**レ**			**レ**	**レ**				
Williams et al. (2017) [50]			**レ**	**レ**	**レ**							
Ylönen et al. (2017) [51]		**レ**		**レ**								
Goodman et al. (2018) [52]				**レ**			**レ**	**レ**				
Meredith et al. (2018) [53]	**レ**	**レ**		**レ**				**レ**		**レ**		
Oneill et al. (2018) [11]			**レ**	**レ**			**レ**	**レ**				
Abel et al.(2019) [54]		**レ**										**レ**(structual empowerment)
Gullatte et al. (2019) [55]												**レ**(capability/comfort)
Kaneko et al. (2019) [56]												**レ**(emotional coping)
Lineker et al. (2019) [57]									**レ**	**レ**		
Schilinski et al. (2019) [58]				**△**								
Colaceci et al. (2020) [59]	**レ**	**レ**		**レ**			**レ**	**レ**	**レ**			
Harvey et al. (2020) [60]	**レ**									**レ**		
Horiguchi et al. (2020) [61]					**レ**							
Howard and Embree (2020) [62]		**レ**		**レ**	**レ**							
Isoyama (2020) [63]				**レ**							**レ**	
Kurotaki (2020) [64]				**レ**			**レ**					
Mun and Hwang (2020) [65]				**レ**								
Shchory et al. (2020) [66]				**レ**				**レ**				
Yeo et al. (2020) [67]			**レ**	**レ**	**レ**							
Yoshida et al. (2020) [68]				**レ**	**レ**							
Lim and Yi (2021) [69]						**レ**						**レ**(awareness)
Matsumoto et al. (2021) [70]				**レ**	**レ**							
Ota (2021) [71]									**レ**			
Williams et al. (2021) [72]				**レ**	**レ**							
Evelyn et al. (2022) [73]				**レ**								
Fang et al. (2022) [74]										**レ**		**レ**(patient outcomes)
Nakamura et al. (2022) [75]				**レ**							**レ**	
Suzuki et al. (2022) [76]		**レ**					**レ**				**レ**	**レ**(self efficacy)
Bos et al. (2023) [77]				**レ**	**レ**					**レ**		

### Modes of delivery

According to Ngenzi et al. (2021) [14], delivery modes can be classified into three categories: *face-to-face or on-campus delivery*, *blended delivery*, and *pure online delivery*. Blended delivery is “a mode of study that encompasses both online and face-to-face learning”, and pure online delivery “encompasses online learning.” Of the 57 studies, 46 used purely online learning, and 11 used blended learning [37, 43, 44, 48, 51, 54, 63, 66, 68, 70, 77].

### Outcomes

As shown in Table 4, all of the included papers reported some outcomes. Knowledge acquisition was the most frequently reported outcome (41 articles), followed by behavior (14 articles) [11, 24, 32, 33, 35, 39, 42, 45, 47, 49, 52, 59, 64, 76], attitude (12 articles) [11, 30, 32, 35, 40, 44, 45, 49, 52, 53, 59, 66], satisfaction (nine articles) [22, 24, 30, 34, 36, 38, 57, 59, 71], and skills (nine articles) [43, 50, 61, 62, 67, 68, 70, 72, 77].

The studies did not use a common method for assessing the outcomes. The following scales and tools were used in the studies: the knowledge, confidence, and attitudes scale [53]; self-efficacy toward helping scale [45]; professional comfort and capability instrument [55]; sexual healthcare practice scale [26]; attitudes regarding the use of restraints scale [44]; and other existing scales as well as independently developed tests, scales, and questionnaires such as comprehension, awareness, and confidence [28, 29, 33, 34, 47–49, 63, 65, 69, 72]. Attitude and behavioral outcome items were previously the main assessment items, but recent years have shown a trend toward assessing skills. In addition, among the enhancing technologies that have been used since 2017, those using computer simulation or virtual reality, scenario-based learning, and gamification were effective in improving knowledge acquisition and actual behavior-related outcomes such as skills, behavior, performance, and attitude [11, 49, 50, 53, 62, 67].

### Benefits of asynchronous e-learning

The benefits of asynchronous e-learning were as follows: cost-effective [25, 30, 32, 39, 47, 57, 70], time-saving and efficient [9, 25, 32, 47, 52, 68, 69, 75, 76], immediate feedback [11, 47, 68], self-paced learning [9, 11, 25, 47, 53, 57, 70, 76], flexibility [50, 70, 72, 75], ease of participation despite location and time limitations [26, 32, 50, 52, 53, 57, 63, 72], ease of participation [30, 35, 51–53, 57, 68], and repeated learning [53].

In addition, the following description of the benefits of the onboard features was provided: the simulation of interactive materials allows for a proxy experience on the screen. The proxy experience enhances self-efficacy, provides motivation, and leads to continued motivation to learn [29]. No one dropped out because the interactive materials kept them engaged [29]; the variety of interactive, multimedia, and hands-on elements helped maintain the nurses' curiosity and interest [43]; fun quiz formats could be used [26]; and innovative and interactive features retained the participants' interest [35]. Regarding motivation, the authors stated that adding incentives increased extrinsic motivation [11], and the connection of learning content to clinical experience increased intrinsic motivation [71]. Creating an active learning experience that promotes a sense of accomplishment among learners to increase motivation is necessary [30].

### Issues related to asynchronous e-learning

The challenges related to asynchronous e-learning were as follows: the need for communication between learners and educators [57, 58]; lack of real-time feedback [71]; the influence of module and evaluation design on learning effectiveness [58]; possible failure to complete the entire module by some participants [58]; lack of time to study lengthy content [26, 51, 62]; lack of computer skills [33, 37, 51]; internet connection problems [9, 33]; lack of follow-up to prevent dropping out [56]; and lack of incentives to stay motivated [26]. Consequently, the need to provide opportunities to observe actual situations [71] has been highlighted, as asynchronous e-learning was considered insufficient for improving confidence [47] and practical skills [22, 71].

The onboard features in gamification present some risks; for instance, in situations involving differences or discontinuities in spatial position and timing of movements between practice with web-based game scenarios and real-life scenarios, game users might negatively modify their performance, and an inappropriate transfer of skills may occur. Moreover, a negative transfer may occur when game users find that the skills they see in the game differ from those needed in real life [67]. It was also stated that research designs to measure the educational effectiveness of e-learning are insufficient because there is a lack of good-quality RCTs to compare the effects of purely digital education [12]. In particular, few studies have measured outcomes on the impact of gamification, and those that have identified educational or clinical outcomes have low power and little clear evidence [67].

## Discussion

Interactive modules and videos are the most frequently used enabling technologies under TEET. In contrast to face-to-face learning or synchronous e-learning, asynchronous e-learning lacks the feeling of being taught directly in real-time. As learners can engage with e-learning modules at their own pace, this may affect their motivation levels. Cheng [79] explored the relationship between intrinsic and extrinsic motivation in nurses’ e-learning. Interaction was one of the most essential factors in the e-learning environment. Based on the Technology Acceptance Model and flow theory, three types of interaction factors (i.e., *learner-system interaction*, *instructor-learner interaction*, and *learner-learner interaction*) that lead to nurses’ acceptance of e-learning systems were studied. The results revealed that instructor–learner interaction was the most crucial antecedent factor impacting nurses’ extrinsic motivation. Notably, learner-learner interaction greatly influences nurses’ intrinsic motivation, suggesting it can promote their learning persistence [79].

The guiding framework for developing asynchronous e-learning modules for healthcare professionals by Sinclair et al. (2017) [80] provides 10 guidelines for creating engaging and effective asynchronous e-learning programs. It states that because many purported e-learning programs have limited interactivity, developing and delivering engaging and pedagogically sound e-learning programs must be based on evidence-based instructional design principles. Interactive elements are required to provide learning guidance, content, and feedback [80]. Therefore, considering the three types of interactions (learner–system, instructor–learner, and learner–learner), using interactivity at the necessary stages can help maintain learners’ motivation.

Video module was the most frequently used enabling technology among the TEET options. Videos and other images can promote a person-centered approach in health professional education and motivate learners by using text, videos, and audio files to introduce them to the “person” under their care. This indicates that by relating to the patient or person who needs nursing care, learners experience a sense of connection and are motivated despite the individualistic asynchronous e-learning method. This method would help learners view their learning as a meaningful engagement with real people [32, 80]. Personal stories are powerful and effective methods for adult learners to retain information [81]. Videos are an effective means of learning because they provide a realistic sense of a particular place or scenario.

Regarding technological enhancement, there was no significant increase in innovations aimed at enhancing internal motivation, but scenario-based learning and gamification are likely to develop further [82]. Innovative e-learning is also being developed to integrate technologies such as virtual reality, virtual patient simulation, and virtual hands-on training to provide activities beyond the linear presentation of information in an e-learning format [1, 12]. These novel e-learning programs are expected to be adopted in clinical education and e-learning in future nursing studies. Using such innovative e-learning methods is expected to benefit knowledge acquisition and the ability of learners to connect what is learned with clinical practice, behavioral change, clinical judgment, and clinical reasoning levels.

The results of the present scoping review also revealed that, in recent years, many studies using video technology and simulation had evaluated skills as outcomes. In the past, the educational effects of e-learning on the continuing professional development of health personnel had focused mainly on learner satisfaction and knowledge acquisition, as the impact on practical behavior change has been considered difficult to assess [80]. There is limited research on evaluating more advanced aspects of education, such as behavioral change and the application of learning in clinical practice [80]. Therefore, there is a need for evaluation metrics or indicators aimed at behavioral change and skill development from innovative e-learning modules [83].

In addition, e-learning and evaluation metrics for TEET options and delivery methods should consider factors that facilitate and inhibit e-learning. Regmi and Jones [10] conducted a systematic review to identify and integrate the facilitating and inhibiting factors influencing e-learning in health sciences education. Their conceptual framework included three broad factors: “*design and delivery*,” “*learning outcomes*,” and “*policy context*” [10]. Because the authors found a clear link between the delivery mechanism of e-learning and the potential learning outcomes, developing asynchronous e-learning modules in light of these three factors can help increase external motivation among nurses.

Our scoping review had some limitations. The TEET modules used for e-learning were not standardized, so we judged based on the text descriptions, which may have caused a selection bias. We may also have missed some functions not detailed in the text. As gray literature was not searched, it is possible that some studies were missed. To grasp a wide range of data on asynchronous e-learning in this scoping review, we included pure online and blended learning. While we extracted content related to asynchronous e-learning in terms of benefits and challenges, the results were likely influenced by the effect of blended learning.

Considering that limiting the scope of this study to nurses would eliminate a wide range of asynchronous e-learning methods, we also expanded the scope to health personnel. However, nurses accounted for a large proportion of the health personnel. As more and more research on asynchronous e-learning is expected to be conducted in the future, subsequent reviews may focus on only nurses to determine the results of the review strategy.

## Conclusions

Using data from the 60 articles on asynchronous e-learning, we categorized the technological approaches, modes of delivery, and outcomes. The most commonly used TEET options were interactive online modules and videos. The popularity of other advanced TEET options, such as computer simulations and gamification, is expected to increase in the future. Furthermore, outcome measures need to be continuously developed, considering that e-learning continues to evolve. Three types of interactions, namely, *learner–system*, *instructor–learner*, and *learner–learner*, are important for asynchronous e-learning. Therefore, using interactive features at necessary stages can help promote motivation among the learners.

## Data Availability

All data generated or analysed during this study are included in this published article.

## Abbreviations

COVID-19: Coronavirus disease 2019
TEET: Technology-enabled and enhanced training
PRISMA-ScR: Preferred Reporting Items for Systematic Reviews, and Meta-Analysis extension for Scoping Reviews
ICT: Information communication technology

## References

[1] Dahlke S, Hunter KF, Amoudu O (2020). Innovation in education with acute care nurses. J Contin Educ Nurs.

[2] Govranos M, Newton JM (2014). Exploring ward nurses’ perceptions of continuing education in clinical settings. Nurse Educ Today.

[3] Seymour-Walsh AE, Bell A, Weber A, Smith T (2020). Adapting to a new reality: COVID-19 coronavirus and online education in the health professions. Rural Remote Health.

[4] Hamilton LS, Grant D, Kaufman JH, Diliberti MK, Schwartz HL, Hunter GP, et al. COVID-19 and the state of K–12 Schools: results and technical documentation from the Spring 2020 American educator panels COVID-19 surveys. https://www.rand.org/pubs/research_reports/RRA168-1.html. Accessed 30 Jan 2023.

[5] Bacher-Hicks A, Goodman J, Mulhern C (2021). Inequality in household adaptation to schooling shocks: Covid-induced online learning engagement in real time. J Public Econ.

[6] Lawn S, Zhi X, Morello A (2017). An integrative review of e-learning in the delivery of self-management support training for health professionals. BMC Med Educ.

[7] Sinclair P, Carstairs M, Shanahan B, Schoch M (2014). The development of a medication calculation competency and quality use of renal medicine e-learning program. Ren Soc Australas J.

[8] Xing W, Ao L, Xiao H, Cheng L, Liang Y, Wang J (2018). Nurses’ attitudes toward, and needs for online learning: differences between rural and urban hospitals in Shanghai, East China. Int J Environ Res Public Health.

[9] Rouleau G, Gagnon MP, Côté J, Payne-Gagnon J, Hudson E, Dubois CA (2019). Effects of e-learning in a continuing education context on nursing care: systematic review of systematic qualitative, quantitative, and mixed-studies reviews. J Med Internet Res.

[10] Regmi K, Jones L (2020). A systematic review of the factors - enablers and barriers - affecting e-learning in health sciences education. BMC Med Educ.

[11] ONeill K, Robb M, Kennedy R, Bhattacharya A, Dominici NR, Murphy A. Mobile technology, just-in-time learning and gamification: innovative strategies for a CAUTI Education Program. Online J Nurs Inform. 2018;22(2). https://www.himss.org/resources/mobile-technology-just-time-learning-and-gamification-innovative-strategies-cauti. Accessed 5 Jul 2023.

[12] Martinengo L, Yeo NJY, Markandran KD, Olsson M, Kyaw BM, Car LT (2020). Digital health professions education on chronic wound management: A systematic review. Int J Nurs Stud.

[13] Scott RE, Maurice M (2014). The spectrum of needed e-Health capacity building - towards a conceptual framework for e-Health ‘training’. Global Telehealth.

[14] Ngenzi JL, Scott RE, Mars M (2021). Information and communication technology to enhance continuing professional development (CPD) and continuing medical education (CME) for Rwanda: a scoping review of reviews. BMC Med Educ.

[15] Button D, Harrington A, Belan I (2014). E-learning & information communication technology (ICT) in nursing education: A review of the literature. Nurse Educ Today.

[16] Peters MD, Marnie C, Tricco AC, Pollock D, Munn Z, Alexander L, et al. Updated methodological guidance for the conduct of scoping reviews. JBI Evid Synth. 2020;18(10):2119–26. 10.11124/JBIES-20-00167. 10.11124/JBIES-20-00167 33038124

[17] Tricco AC, Lillie E, Zarin W, O'Brien KK, Colquhoun H, Levac D (2018). PRISMA extension for scoping reviews (PRISMA-ScR): checklist and explanation. Ann Intern Med.

[18] Arksey H, O'Malley L (2005). Scoping studies: towards a methodological framework. Int J Soc Res Methodol.

[19] Levac D, Colquhoun H, O’Brien KK (2010). Scoping studies: advancing the methodology. Implement Sci.

[20] Peters MDJ, Godfrey C, McInerney P, Baldini Soares C, Khalil H, Parker D. Scoping reviews In: Aromataris E. Munn Z, eds. Joanna Briggs Institute Reviewer’s Manual. Adelaide, Australia: Joanna Briggs Inst: 2020.https://reviewersmanual. Joannabriggs.org/. Accessed 30 Jan 2023.

[21] Dennison HA (2011). Creating a computer-assisted learning module for the non-expert nephrology nurse. Nephrol Nurs J.

[22] Sherriff K, Burston S, Wallis M (2012). Effectiveness of a computer based medication calculation education and testing programme for nurses. Nurs Educ Today.

[23] Spiva L, Johnson K, Robertson B, Barrett DT, Jarrell NM, Hunter D (2012). The effectiveness of nurses’ ability to interpret basic electrocardiogram strips accurately using different learning modalities. J Contin Educ Nurs.

[24] Gordon JS, Mahabee-Gittens EM, Andrews JA, Christiansen SM, Byron DJ (2013). A randomized clinical trial of a web-based tobacco cessation education program. Pediatrics.

[25] Alipour S, Jannat F, Hosseini L (2014). Teaching breast cancer screening via text messages as part of continuing education for working nurses: A case-control study. Asian Pac Cancer Prev.

[26] Kim JH, Shin JS (2014). Effects of an online problem-based learning program on sexual health care competencies among oncology nurses: a pilot study. J Contin Educ Nurs.

[27] Liu W-I, Chu K-C, Chen S-C (2014). The development and preliminary effectiveness of a nursing case management e-learning program. Comput Inform Nurs.

[28] McCrow J, Sullivan KA, Beattie ER (2014). Delirium knowledge and recognition: a randomized controlled trial of a web-based educational intervention for acute care nurses. Nurs Educ Today.

[29] Yoshikawa Y (2014). Comparative study of the e-learning materials evaluation in newcomer nursing education: Comparative to interactive teaching materials and video teaching materials. Int Nurs Care Res.

[30] De Gagne JC, Park S, So A, Wu B, Palmer MH, McConnell ES (2015). A urinary incontinence continuing education online course for community health nurses in South Korea. J Contin Educ Nurs.

[31] Delaney MM, Friedman MI, Dolansky MA, Fitzpatrick JJ (2015). Impact of a sepsis educational program on nurse competence. J Contin Educ Nurs.

[32] Hsu TC, Chiang-Hanisko L, Lee-Hsieh J, Lee GY, Turton MA, Tseng YJ (2015). Effectiveness of an online caring curriculum in enhancing nurses’ caring behavior. J Contin Educ Nurs.

[33] Johnson M, Kelly L, Siric K, Tran DT, Overs B (2015). Improving falls risk screening and prevention using an e-learning approach. J Nurs Manag.

[34] Kato C, Katoka Y, Igarashi Y, Hiruta A (2015). Evaluation of an e-learning program of continuing midwifery education on postpartum hemorrhage. J Jpn Acad Midwif.

[35] Murphy J, Worswick L, Pulman A, Ford G, Jeffery J (2015). Translating research into practice: evaluation of an e-learning resource for health care professionals to provide nutrition advice and support for cancer survivors. Nurse Educ Today.

[36] Sarabia-Cobo CM, Torres-Manrique B, Ortego-Mate MC, Salvadores-Fuentes P, Sáenz-Jalón M (2015). Continuing education in patient safety: Massive open online courses as a new training tool. J Contin Educ Nurs.

[37] Berggren E, Orrevall Y, Olin AÖ, Strang P, Szulkin R, Törnkvist L (2016). Evaluation of a continuing educational intervention for primary health care professionals about nutritional care of patients at home. J Nutr Health Aging.

[38] Okuroğlu GK, Alpar ŞE (2016). Development of a web-based diabetes education program for health care professionals. J Contin Educ Nurs.

[39] Perrego K (2017). Improving staff knowledge of perioperative regulated-waste management. AORN J.

[40] Sarna L, Bialous SA, Zou XN, Wang W, Hong J, Wells M (2016). Evaluation of a web-based educational programme on changes in frequency of nurses’ interventions to help smokers quit and reduce second-hand smoke exposure in China. J Adv Nurs.

[41] Sinclair PM, Kable A, Levett-Jones T, Booth D (2016). The effectiveness of Internet-based e-learning on clinician behaviour and patient outcomes: A systematic review. Int J Nurs Stud.

[42] Bond SE, Crowther SP, Adhikari S, Chubaty AJ, Yu P, Borchard JP (2017). Design and implementation of a novel web-based e-learning tool for education of health professionals on the antibiotic vancomycin. J Med Internet Res.

[43] Glover KR, Stahl BR, Murray C, LeClair M, Gallucci S, King MA (2017). A simulation-based blended curriculum for short peripheral intravenous catheter insertion: an industry-practice collaboration. J Contin Educ Nurs.

[44] Kong EH, Song E, Evans LK (2017). Effects of a multicomponent restraint reduction program for Korean nursing home staff. J Nurs Scholarsh.

[45] Manning JC, Carter T, Latif A, Horsley A, Cooper J, Armstrong M (2017). ‘Our Care through Our Eyes’. Impact of a co-produced digital educational programme on nurses’ knowledge, confidence and attitudes in providing care for children and young people who have self-harmed: A mixed-methods study in the UK. BMJ Open.

[46] Micheel CM, Anderson IA, Lee P, Chen SC, Justiss K, Giuse NB (2017). Internet-based assessment of oncology health care professional learning style and optimization of materials for web-based learning: Controlled trial with concealed allocation. J Med Internet Res.

[47] Shin JY, Issenberg SB, Roh YS (2017). The effects of neurologic assessment e-learning in nurses. Nurse Educ Today.

[48] Smith JM, Van Aman MN, Schneiderhahn ME, Edelman R, Ercole PM (2017). Assessment of delirium in intensive care unit patients: educational strategies. J Contin Educ Nurs.

[49] Trudeau KJ, Hildebrand C, Garg P, Chiauzzi E, Zacharoff KL (2017). A randomized controlled trial of the effects of online pain management education on primary care providers. Pain Med.

[50] Williams K, Abd-Hamid NH, Perkhounkova Y (2017). Transitioning communication education to an interactive online module format. J Contin Educ Nurs.

[51] Ylönen M, Viljamaa J, Isoaho H, Junttila K, Leino-Kilpi H, Suhonen R (2017). Internet-based learning programme to increase nurses’ knowledge level about venous leg ulcer care in home health care. J Clin Nurs.

[52] Goodman HA, Pacheco CL, Loescher LJ (2018). an online intervention to enhance nurse practitioners' skin cancer knowledge, attitudes, and counseling behaviors: a pilot study. J Dermatol Nurs Assoc.

[53] Meredith P, Yeates H, Greaves A, Taylor M, Slattery M, Charters M (2018). Preparing mental health professionals for new directions in mental health practice: Evaluating the sensory approaches e-learning training package. Int J Ment Health Nurs.

[54] Abel SE, Hall M, Swartz MJ, Madigan EA (2020). Empowerment of front-line leaders in an online learning, certificate programme. J Nurs Manag.

[55] Gullatte MM, Allen CS, Botheroyd E, Hess RG, Higgins M, Meneghetti J (2019). Improving end-of-life communications using technology-assisted continuing education with interprofessional teams. J Nurs Prof Dev.

[56] Kaneko T, Morita N, Ito M, Sekiya D (2019). Examination of the impact of the educational web program to improve emotional coping with emotional labor among nurses. J Jpn Acad Nurs Sci.

[57] Lineker SC, Fleet LJ, Bell MJ, Sweezie R, Curran V, Brock G (2019). Getting a grip on arthritis online: responses of rural/remote primary care providers to a web-based continuing medical education programme. Can J Rural Med.

[58] Schilinski S, Hellier SD, Cline TW (2019). Evaluation of an electronically delivered learning module intended for continuing education of practicing registered nurses: a pretest-posttest longitudinal study. J Contin Educ Nurs.

[59] Colaceci S, Zambri F, D’amore C, De Angelis A, Rasi F, Pucciarelli G (2020). Long-term effectiveness of an e-learning program in improving health care professionals’ attitudes and practices on breastfeeding: A 1-year follow-up study. Breastfeed Med.

[60] Harvey A, Zhang Y, Phillips S, Suarez R, Dekle L, Villalobos A (2020). Initial outcomes of an online continuing education series focused on post-treatment cancer survivorship care. J Cancer Educ.

[61] Horiguchi T, Asada Y, Tasaki K, Inagaki M (2020). Investigation of educational methods using e-learning teaching materials to improve nurses’ skills in promoting team-based diabetes medical care. J Jpn Acad Nurs Sci.

[62] Howard MS, Embree JL (2020). Educational intervention improves communication abilities of nurses encountering workplace incivility. J Contin Educ Nurs.

[63] Akemi I (2020). Development and assessment of a training program for improving midwives’ knowledge and perceptions of family role acquisition in the perinatal period. J Jpn Acad Midwif.

[64] Kurotaki A (2020). The effect of an educational program on improving the ability of hospital nursing administrators to accept supporting nurses. J Japan Soc Disaster Nurs.

[65] Mun MY, Hwang SY (2020). Development and evaluation of a web-based learning course for clinical nurses: anticancer chemotherapy and nursing. Korean J Adult Nurs.

[66] Shchory MP, Goldstein LH, Arcavi L, Shihmanter R, Berkovitch M, Levy A (2020). The effect of an intervention program on the knowledge and attitudes among medical staff regarding adverse drug reaction reporting. Pharmacoepidemiol Drug Saf.

[67] Yeo CL, Ho SKY, Tagamolila VC, Arunachalam S, Bharadwaj SS, Poon WB (2020). Use of web-based game in neonatal resuscitation - is it effective?. BMC Med Educ.

[68] Yoshida M, Miura Y, Yabunaka K, Sato N, Matsumoto M, Yamada M (2020). Efficacy of an education program for nurses that concerns the use of point-of-care ultrasound to monitor for aspiration and pharyngeal post-swallow residue: A prospective, descriptive study. Nurse Educ Pract.

[69] Lim H, Yi Y (2021). Effects of a web-based education program for nurses using medical malpractice cases: A randomized controlled trial. Nurse Educ Today.

[70] Matsumoto M, Tamai N, Miura Y, Okawa Y, Yoshida M, Igawa Y (2021). Evaluation of a point-of-care ultrasound educational program for nurse educators. J Contin Educ Nurs.

[71] Ota Y (2021). Development and operation of an e-learning program for nurses to support attachment formation between mother and child in the early postpartum period. Matern Health.

[72] Williams KN, Coleman CK, Perkhounkova Y, Beachy T, Hein M, Shaw CA (2021). Moving online: A pilot clinical trial of the changing talk online communication education for nursing home staff. Gerontologist.

[73] Evelyn AE, Kittelson S, Mandernach MW, Black V, Duckworth L, Wilkie DJ (2022). Nursing education for the acute care nurse on pain mechanisms of sickle cell disease. J Contin Educ Nurs.

[74] Fang J, Chen S, Yang L, Liao K, Lin C, Fujimori M (2022). Improving transitional care through online communication skills training. Aging Clin Exp Res.

[75] Nakamura S, Takeuchi S, Hoshino T, Okubo N, Horiuchi S (2022). Effects of web-based learning for nurses on their care for pregnant women with hiesho (sensitivity of hands or feet to cold): A randomized controlled trial. Jpn J Nurs Sci.

[76] Suzuki M, Yoshimura H, Omuro S, Sawaki K, Naito T, Inagaki K (2022). Effectiveness of a dementia nursing practice skills development program for nurses in acute care hospitals. Jpn J Geriatr.

[77] Bos–van den Hoek DW, Smets EMA, Ali R, Baas-Thijssen MCM, Bomhof-Roordink H, Helsper CW (2023). A blended learning for general practitioners and nurses on skills to support shared decision-making with patients about palliative cancer treatment: A one-group pre-posttest study. Patient Educ Couns.

[78] Hsieh H-F, Shannon SE (2005). Three approaches to qualitative content analysis. Qual Health Res.

[79] Cheng YM (2013). Exploring the roles of interaction and flow in explaining nurses’ e-learning acceptance. Nurse Educ Today.

[80] Sinclair PM, Levett-Jones T, Morris A, Carter B, Bennett PN, Kable A (2017). High engagement, high quality: A guiding framework for developing empirically informed asynchronous e-learning programs for health professional educators. Nurs Health Sci.

[81] Prusak L, Groh K, Denning S, Brown JS (2016). Storytelling in Organizations: Why Storytelling is Transforming 21st Century Organizations and Management.

[82] Bezovski Z, Poorani S (2016). The evolution of e-learning and new trends. Inf Knowl Manag.

[83] Webb TL, Joseph J, Yardley L, Michie S (2010). Using the internet to promote health behavior change: a systematic review and meta-analysis of the impact of theoretical basis, use of behavior change techniques, and mode of delivery on efficacy. J Med Internet Res.

